# Supplementary material for: Role of Non-Coding Regulatory Elements in the Control of GR-Dependent Gene Expression
Source: Int J Mol Sci. 2021 Apr 20;22(8):4258. doi: 10.3390/ijms22084258 (PMC8073421; doi:10.3390/ijms22084258)
Supplement: Supplementary file 1 [file ijms-22-04258-s001.zip › Supplementary Role of non-coding regulatory elements in control of GR-dependent gene expression-M.Z..pdf]

## Supplementary material

# Role of non-coding regulatory elements in the control of GR-dependent gene expression

Malgorzata Borczyk*, Mateusz Zieba*, Michał Korostyński, Marcin Piechota
Laboratory of Pharmacogenomics, Department of Molecular Neuropharmacology, Maj Institute of Pharmacology, Polish Academy of Sciences, Krakow, Poland
*equal contribution

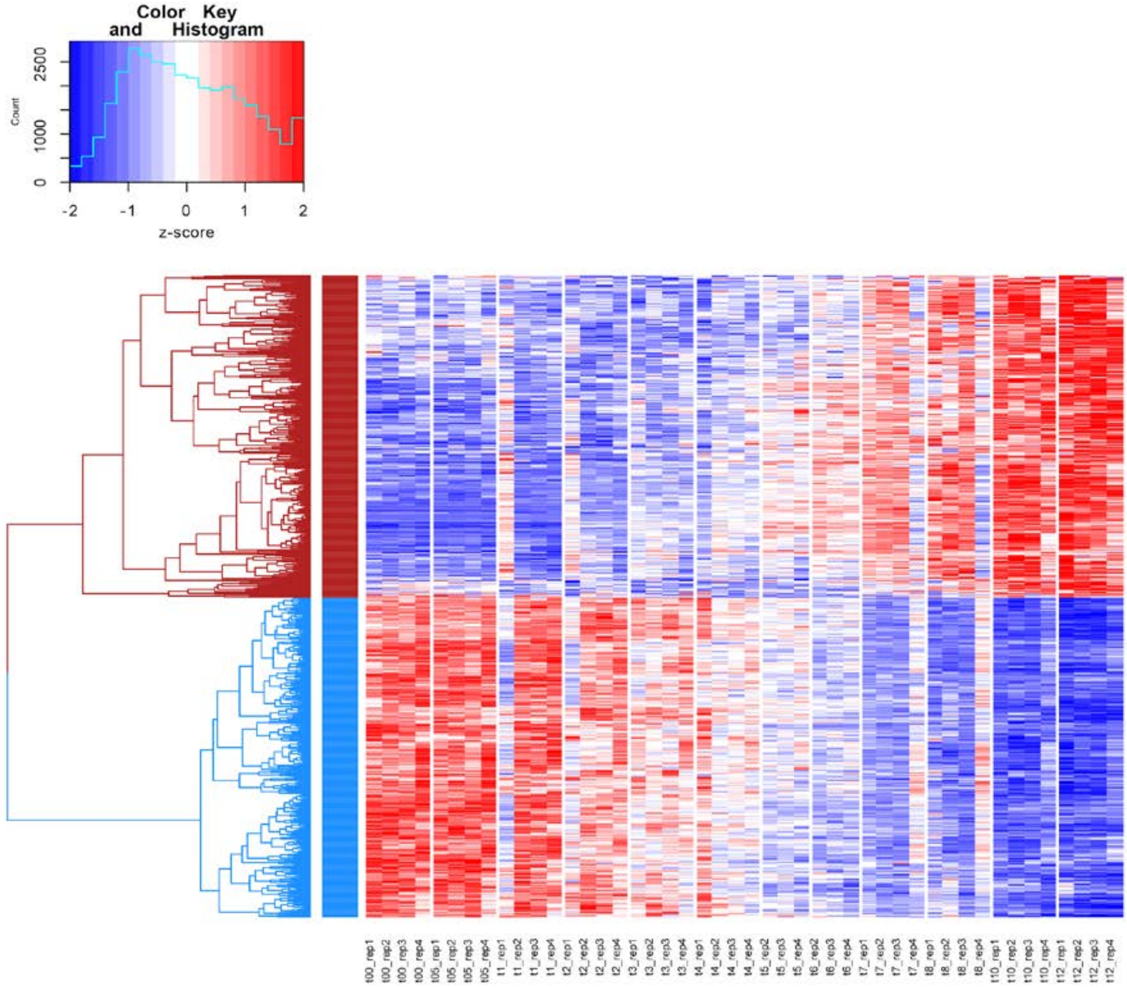


Figure S1 Heatmap of gene expression of selected differentially-expressed genes

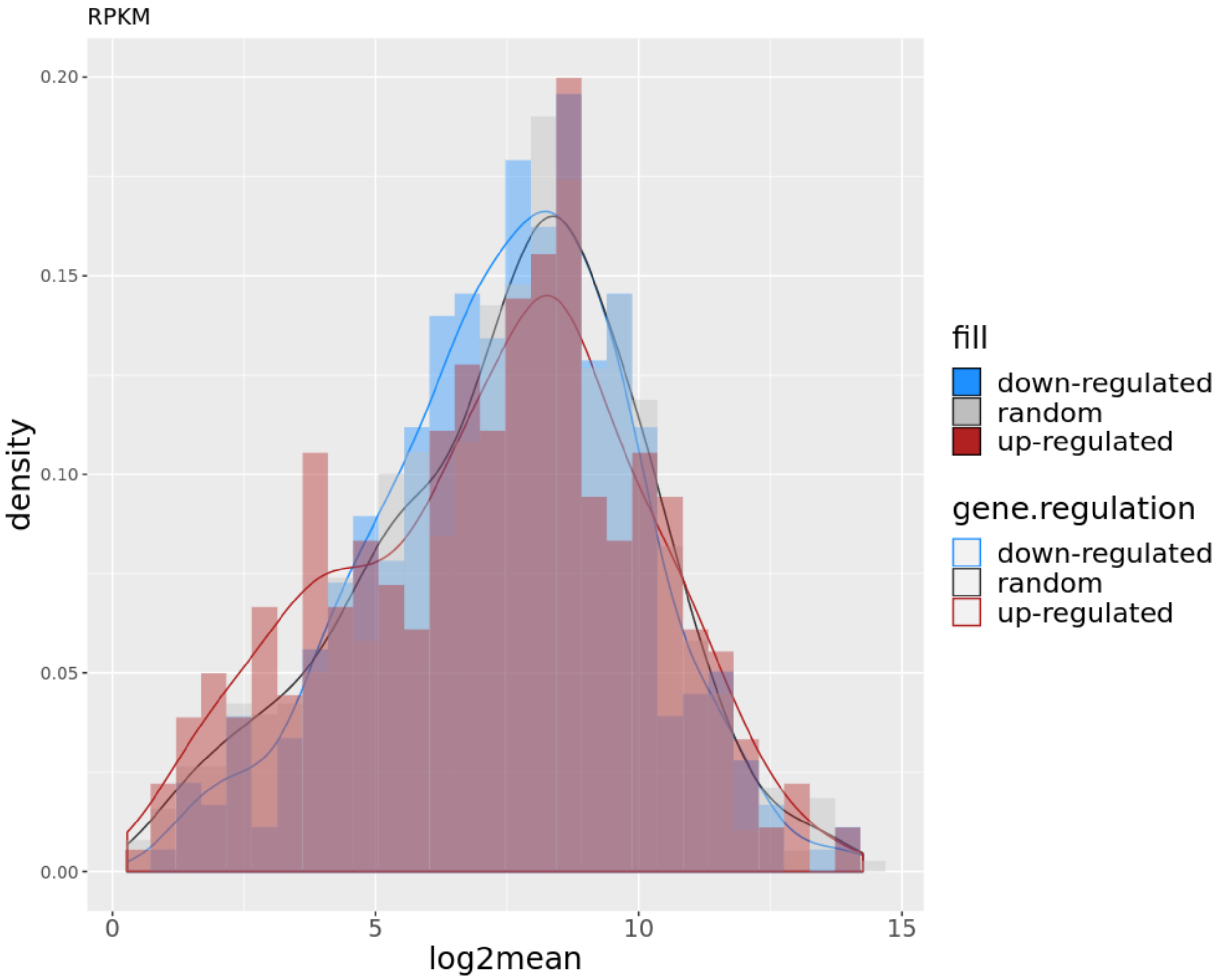


Figure S2 Mean abundance (RPKM) histogram of analysed genes. In order to investigate whether there are any intrinsic differences in the average abundance of investigated transcripts for each transcript the normalised abundance (log2 (mean RPKM)) was calculated and averaged between all the time points. These values were then collected into a histogram and compared between each of the three clusters (up, down and random). x-axis: log2 of mean gene transcript count (RPMK), y-axis: fraction of transcripts in each bin (out of 1).

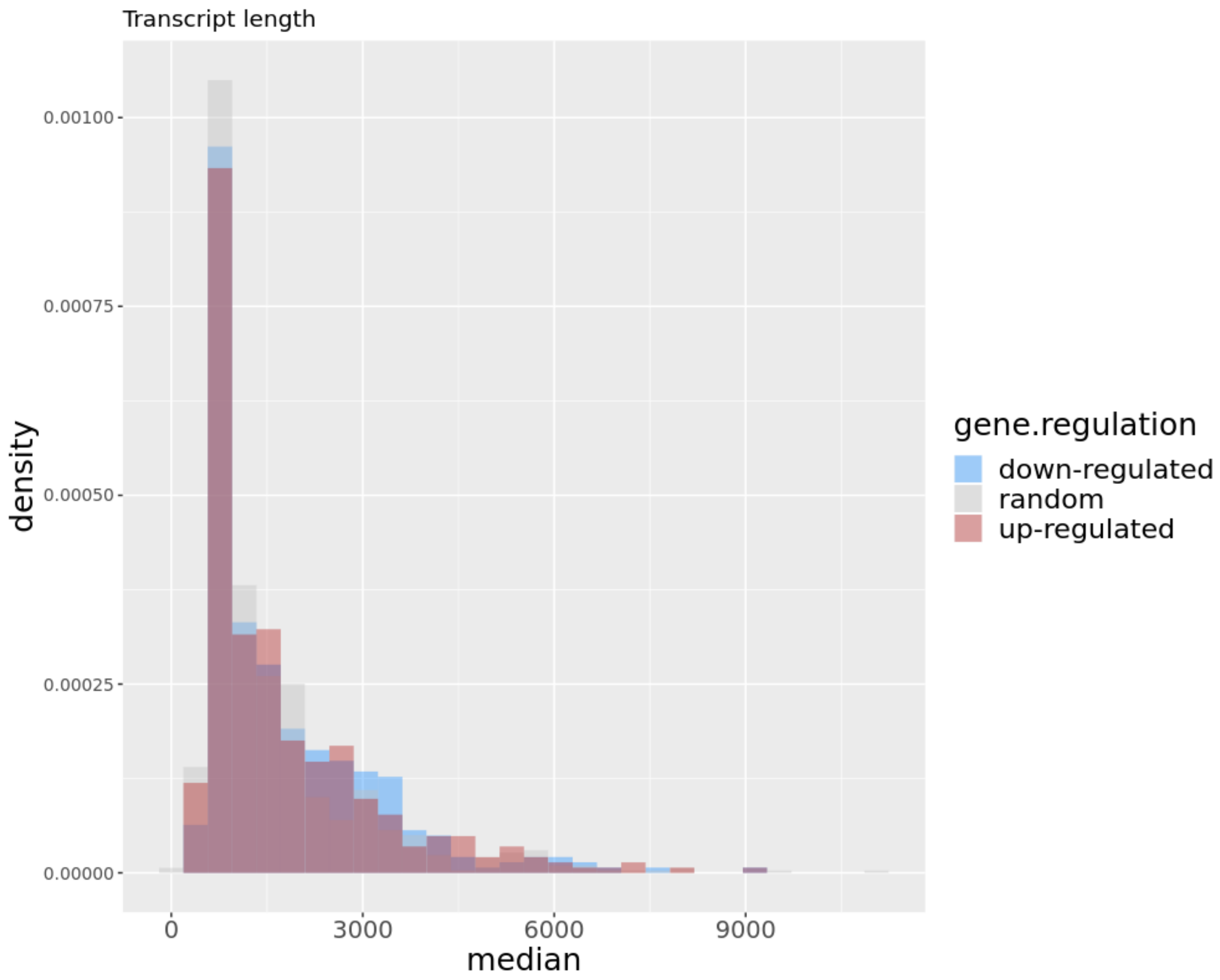


Figure S3 transcript length histogram for analysed genes. In case of multiple transcripts the median transcript length was used.

Table S1 Results of ANOVA on gene expression with the full list of regulated genes together with the raw data, p-value, FDR and cluster information

Table S2 Results of ANOVA on gene expression with the full list of randomly selected genes together with the raw data, p-value, FDR and cluster information.

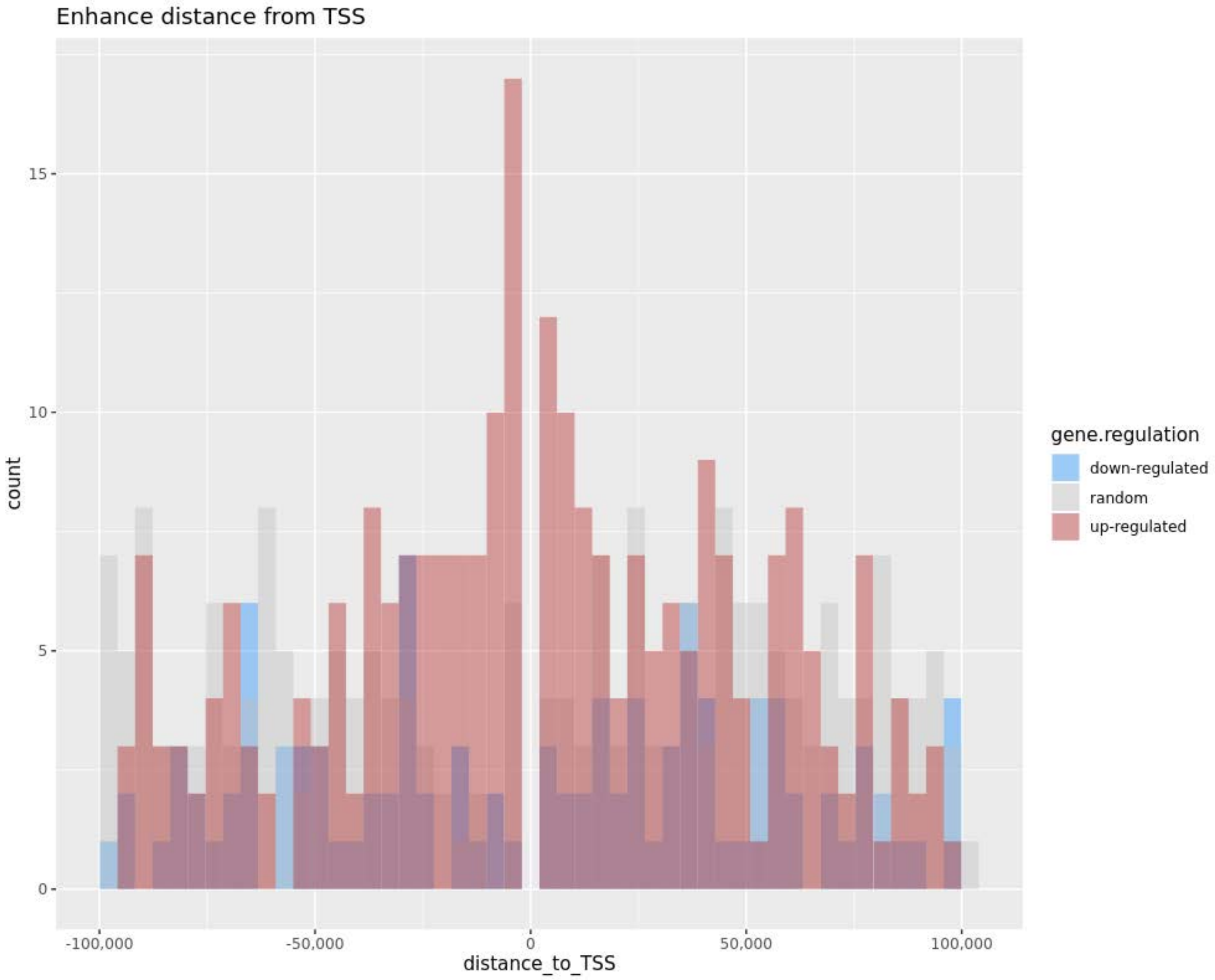


Figure S4 Histogram of GR strongest enhancer peak amplitude distance from transcription start site (TSS). For each of the differentially regulated genes a single NR3C1 binding site in an enhancer region of each gene was chosen for analysis. This single site was based on the strongest (highest amplitude) NR3C1 peak at the 60 min time point. Enhancer regions were determined as located between -100 to -2 kb and +2 to +100 kb from TSS (the core promoter region was excluded). y-axis represents distance from TSS in bp; x-axis - number of peaks in each 4 kb bin. Peaks from each of the clusters: up, down and random are summarised separately.

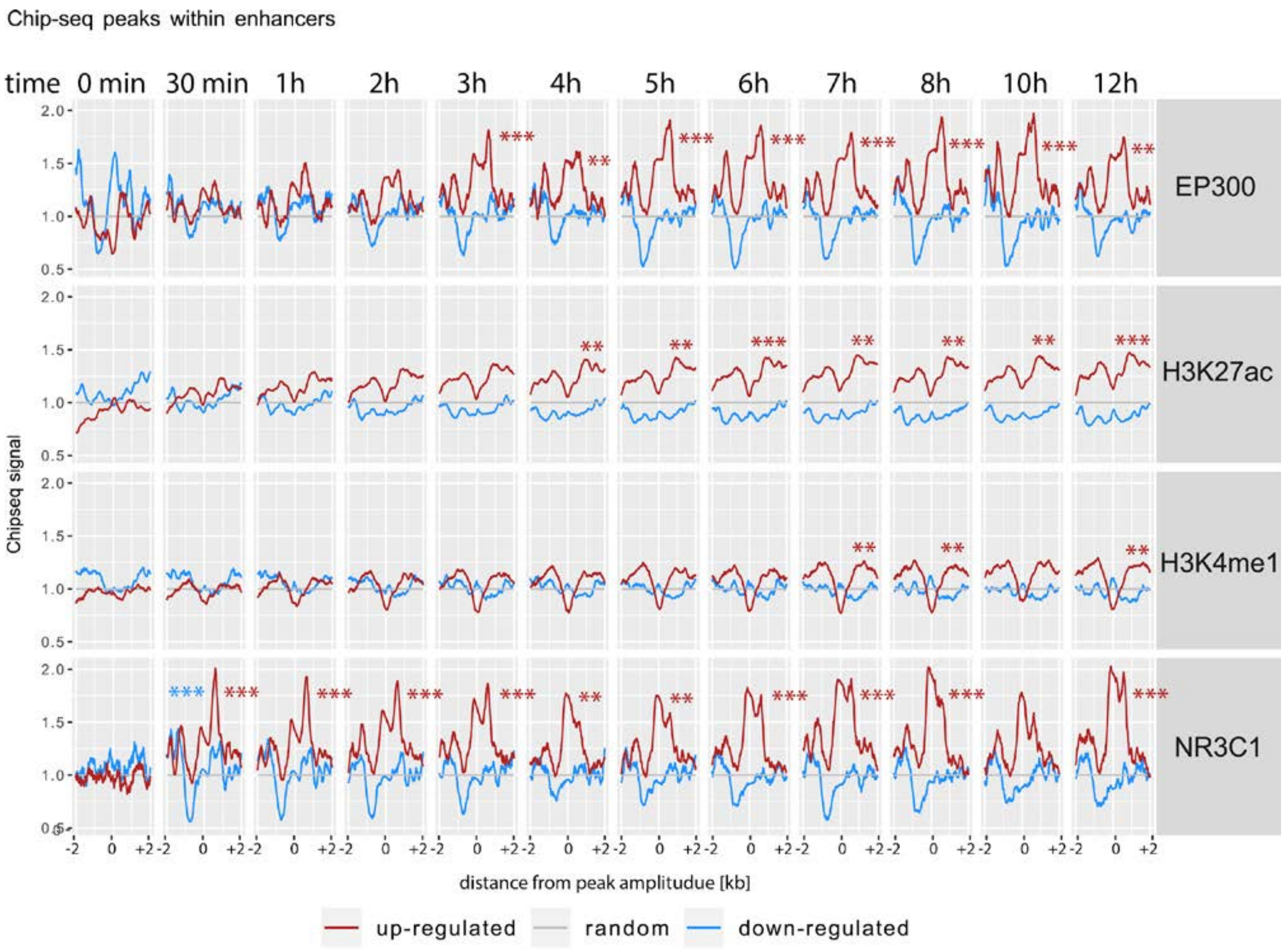


Figure S5 Line-plot normalised to random genes for enhancer peaks

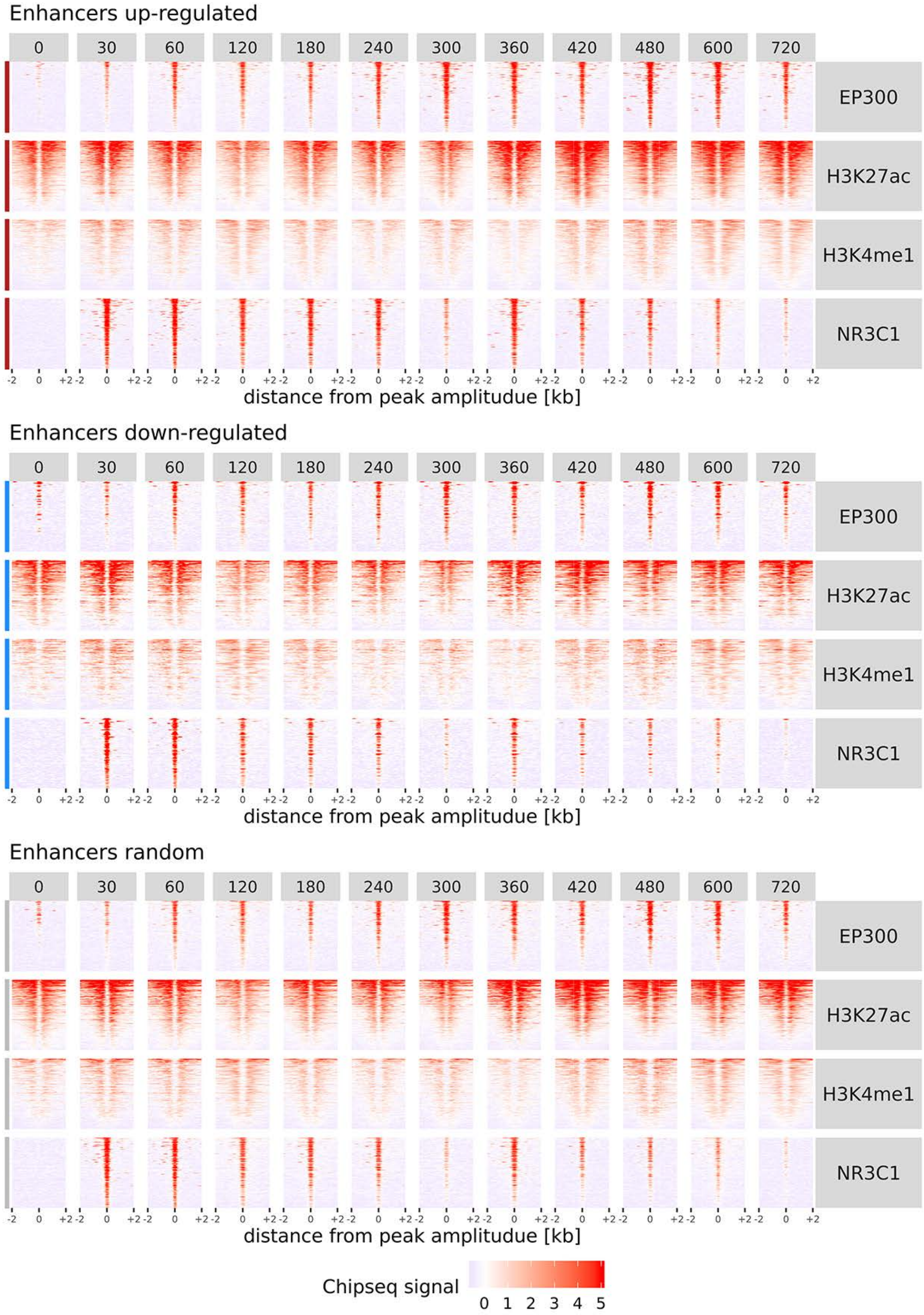


Figure S6 Heatmap of data presented in Figure 2

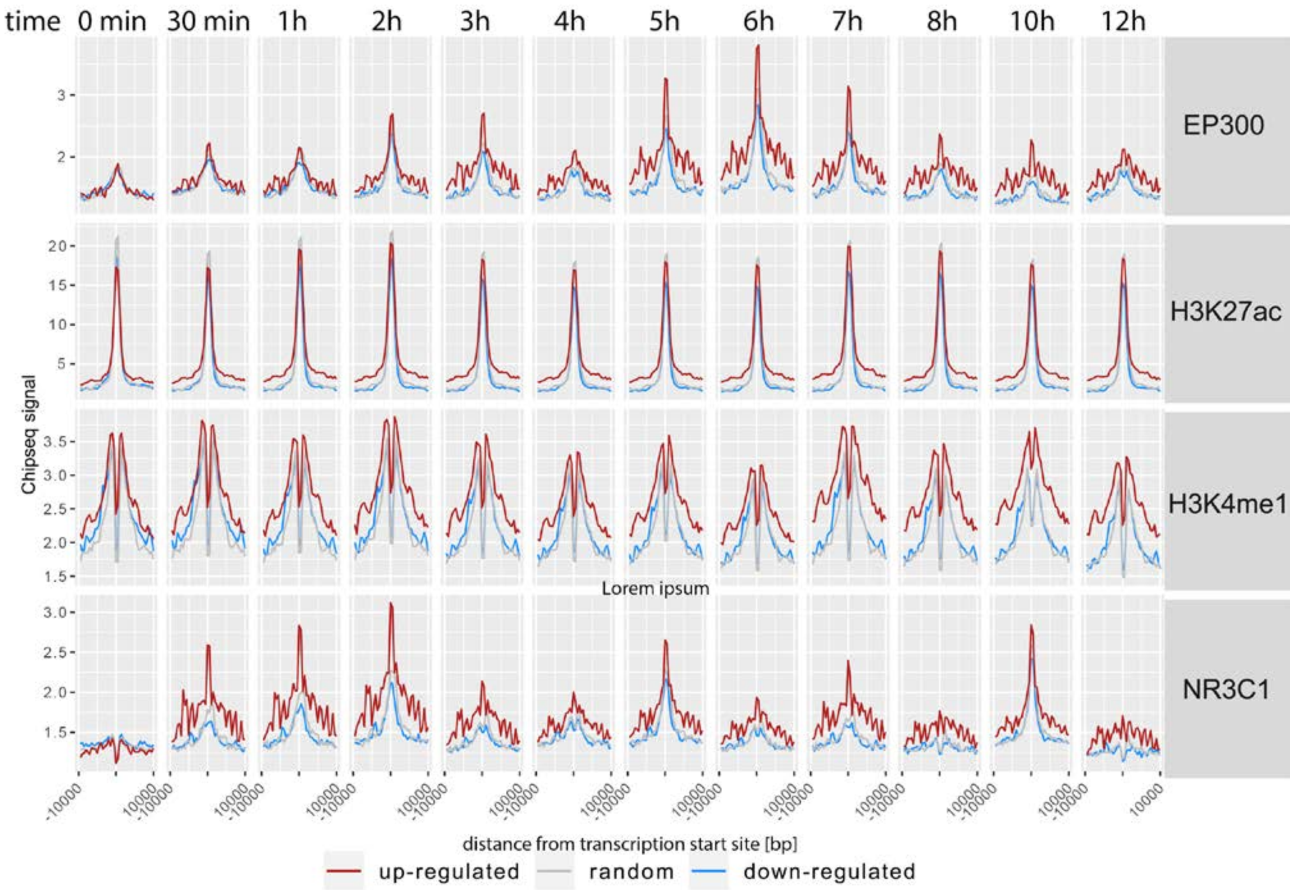


Figure S7 Line-plot for promotor peaks (axes +/- 10 000 from TSS). Here peaks were not centereds, but simply average signal was plotted for the chosen interval.

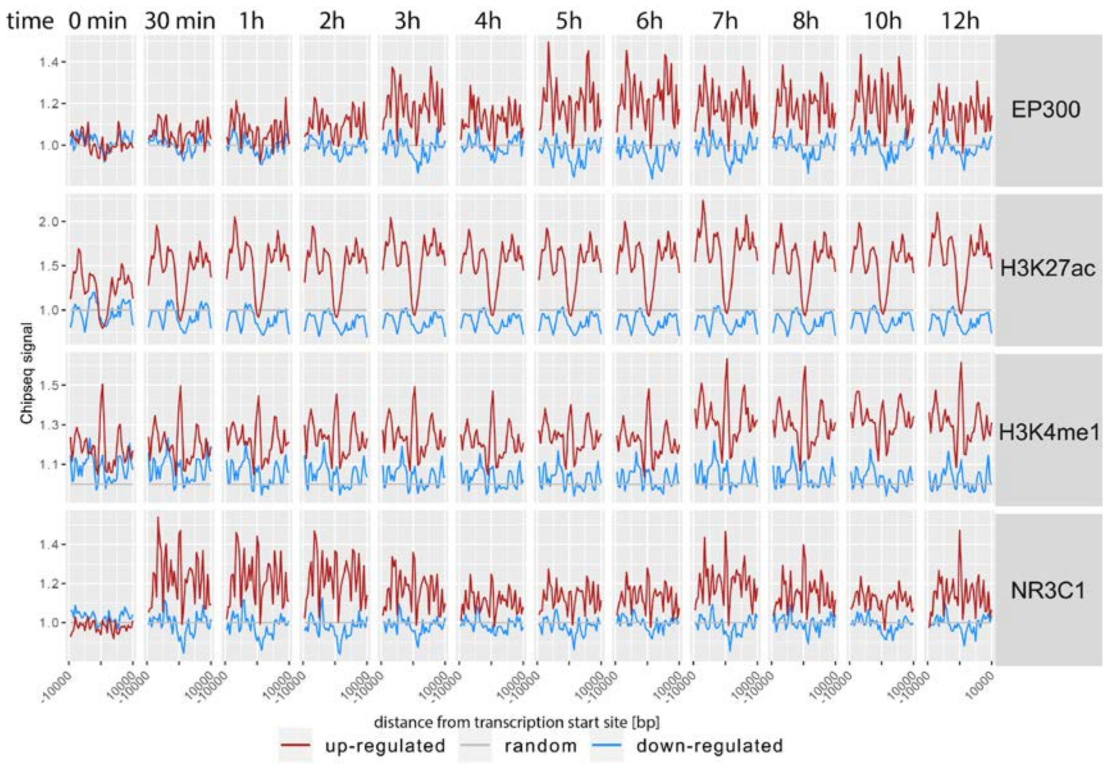


Figure S8 Line-plot normalised to random genes for promotor peaks (axes +/- 10 000 from TSS)

Table S3 Two-way ANOVA (time x gene regulation cluster) results based on amplitudes of normalised peaks (data presented in Figure S2.2)

Table S4 Post-hoc pairwise t-tests of amplitudes of normalised peaks , comparisons between each timepoint ant 0 min

Table S5 Post-hoc pairwise t-tests of amplitudes of normalised peaks ., comparisons between upregulated and downregulated genes

Table S6 one-way ANOVA results of weighted timepoints (data presented in Figure 3A)

Table S7 Post-hoc results of weighted timepoints (data presented in Figure 3A)

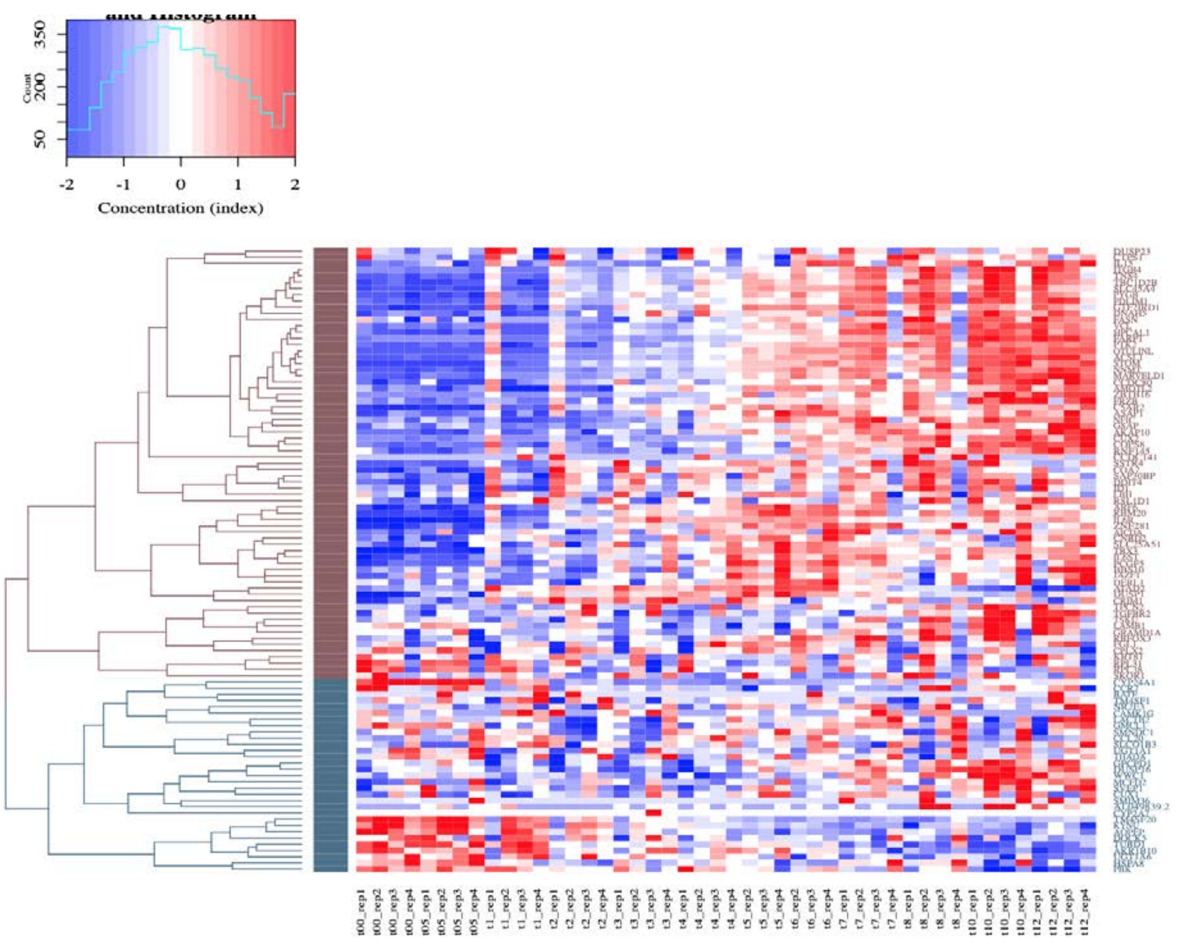


Figure S9 Heatmap of gene expression of genes with highest EP300 changes

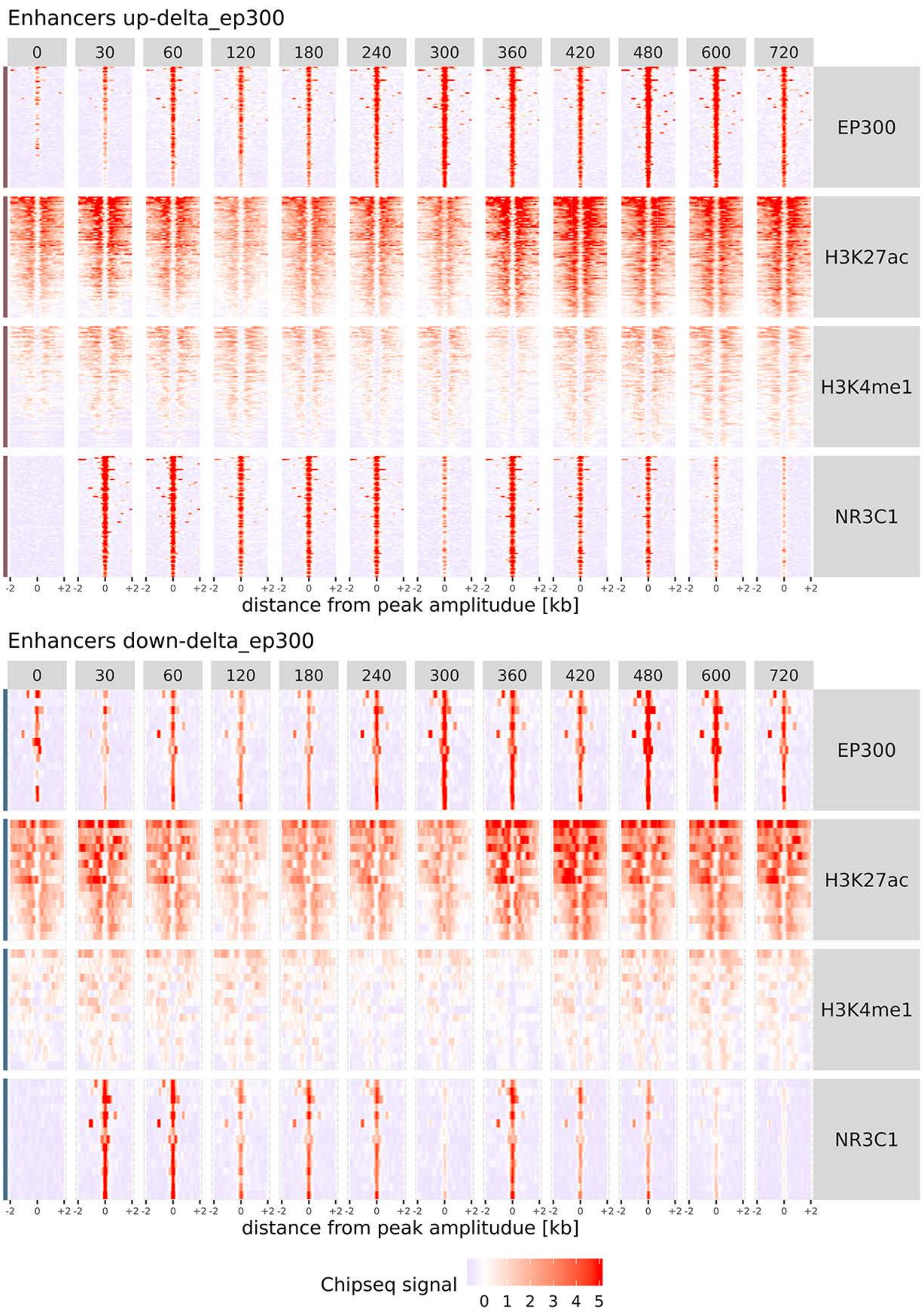


Figure S10 Heatmap of gene ChIP-seq peaks for genes with highest EP300 changes

Table S8 two-way ANOVA results of weighted timepoints presented in Figure 4

Table S9 Post-hoc results of weighted timepoints presented in Figure 3A
